# Supplementary material for: Impact of MAOA Gene Polymorphism on the Efficacy of Antidepressant Treatment and Craving Severity for Betel Quid Use Disorder
Source: Int J Mol Sci. 2024 Aug 25;25(17):9221. doi: 10.3390/ijms25179221 (PMC11394840; doi:10.3390/ijms25179221)
Supplement: Supplementary file 1 [file ijms-25-09221-s001.zip › 1_ijms-3159688-Supplementary.docx]

**Supplementary figures:**

**Interaction between MAOA genotypes and follow-up Yales-Brown Compulsive Obsessive Scale-betel-quid**


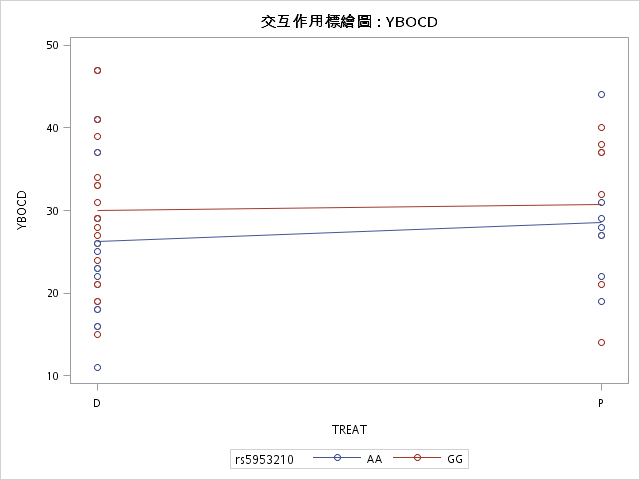


Figure S1. Interaction between MAOA genotypes and follow-up Yales-Brown Compulsive Obsessive Scale-betel-quid (Week 0)


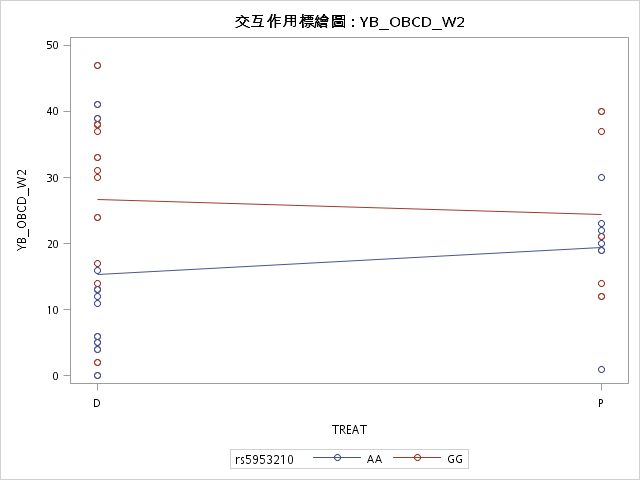


Figure S2. Interaction between MAOA genotypes and follow-up Yales-Brown Compulsive Obsessive Scale-betel-quid (follow-up time: Week 2)


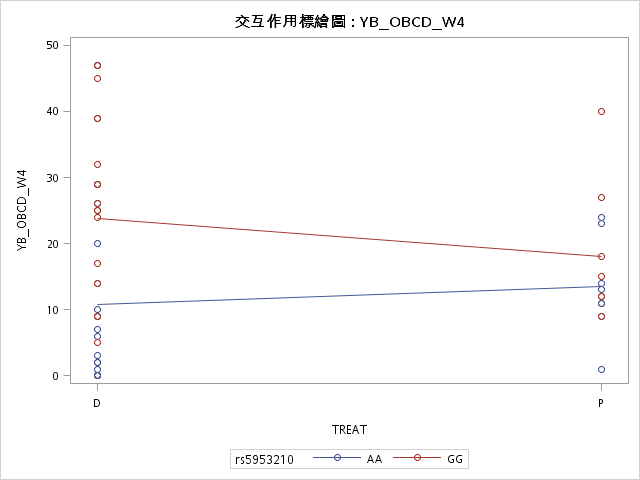


Figure S3. Interaction between MAOA genotypes and follow-up Yales-Brown Compulsive Obsessive Scale-betel-quid (follow-up time: Week 4)


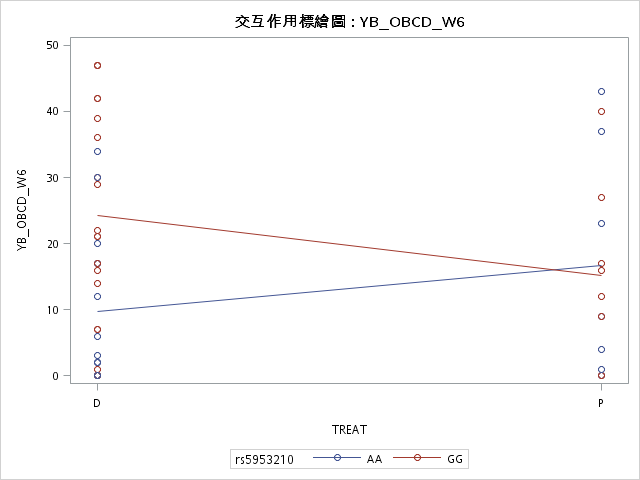


Figure S4. Interaction between MAOA genotypes and follow-up Yales-Brown Compulsive Obsessive Scale-betel-quid (follow-up time: Week 6)


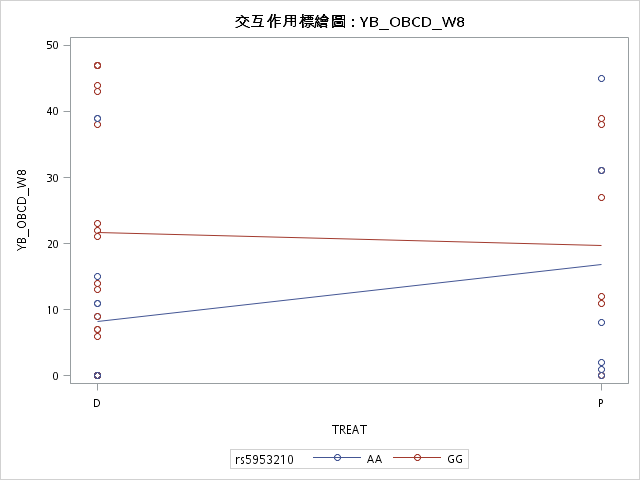


Figure S5. Interaction between MAOA genotypes and follow-up Yales-Brown Compulsive Obsessive Scale-betel-quid (follow-up time: Week 8)
